# Adsorption and separations of small molecules in porous media

**DOI:** 10.1063/4.0001029

**Published:** 2025-10-27

**Authors:** Craig M Brown

**Affiliations:** NIST Center for Neutron Research, Gaithersburg, MD, USA

## Abstract

Metal-organic frameworks (MOFs) are crystalline materials that contain metal-ions or metal-ion clusters as nodes and organic ligands as linkers to form 0-, 1-, 2-, and 3-D structures. Their structural versatility and multifunctional properties have sparked much interest in advanced materials synthesis, and due to their modular nature, many of these materials can be constructed by design. Over the last decade, several MOFs reportedly have high surface areas, allowing them to physically adsorb significant amounts of gas and/or exhibit significant separation performance. The adsorption of molecules in functionalized and high-surface microporous materials is of technological importance in many areas, ranging from catalysis, drug delivery, chemical separations, and energy storage to personal care products. Their uptake properties can be tuned by carefully selecting the ligand and metal that control pore size/shape and MOF-adsorbate interactions. We have focused on understanding the properties of gas interactions within various microporous materials to identify and understand new optimal storage and separation materials. We have recently structurally characterized high-capacity room-temperature hydrogen storage in MOFs through careful ligand and exposed metal cation availability. [1][2] We can further delineate ligand dynamic impact on controlling kinetic-to-thermodynamic control regimes for selective gas adsorption in the simplest ultra-microporous MOF, aluminum formate. This allows the temperature-controlled separations of H__2__, Ar, O__2__, CO_2_, and C_2_H_2_ from such gases as N_2_ and CH_4_. [3][4][5][6] Our most recent work details the mechanism for high-temperature (300 ^o^C) capture of CO_2_ from a wet gas stream in a metal-H containing MOF. We use in-situ synchrotron X-ray powder diffraction to illustrate the quantitive formate formation upon adsorption and identify the hydride by applying neutron powder diffraction. [7] We will discuss these recent results in context and illustrate the potential for industrial uses of MOFs.
